# Supplementary material for: A Global Screen for Assembly State Changes of the Mitotic Proteome by SEC-SWATH-MS
Source: Cell Syst. 2020 Feb 26;10(2):133–155.e6. doi: 10.1016/j.cels.2020.01.001 (PMC7042714; doi:10.1016/j.cels.2020.01.001)

Q9BZG8 | DPH1_HUMAN | DPH1 DPH2L DPH2L1 OVCA1
Monomer MW [kDa]: 48.805 Monomer expected elution fraction: 46

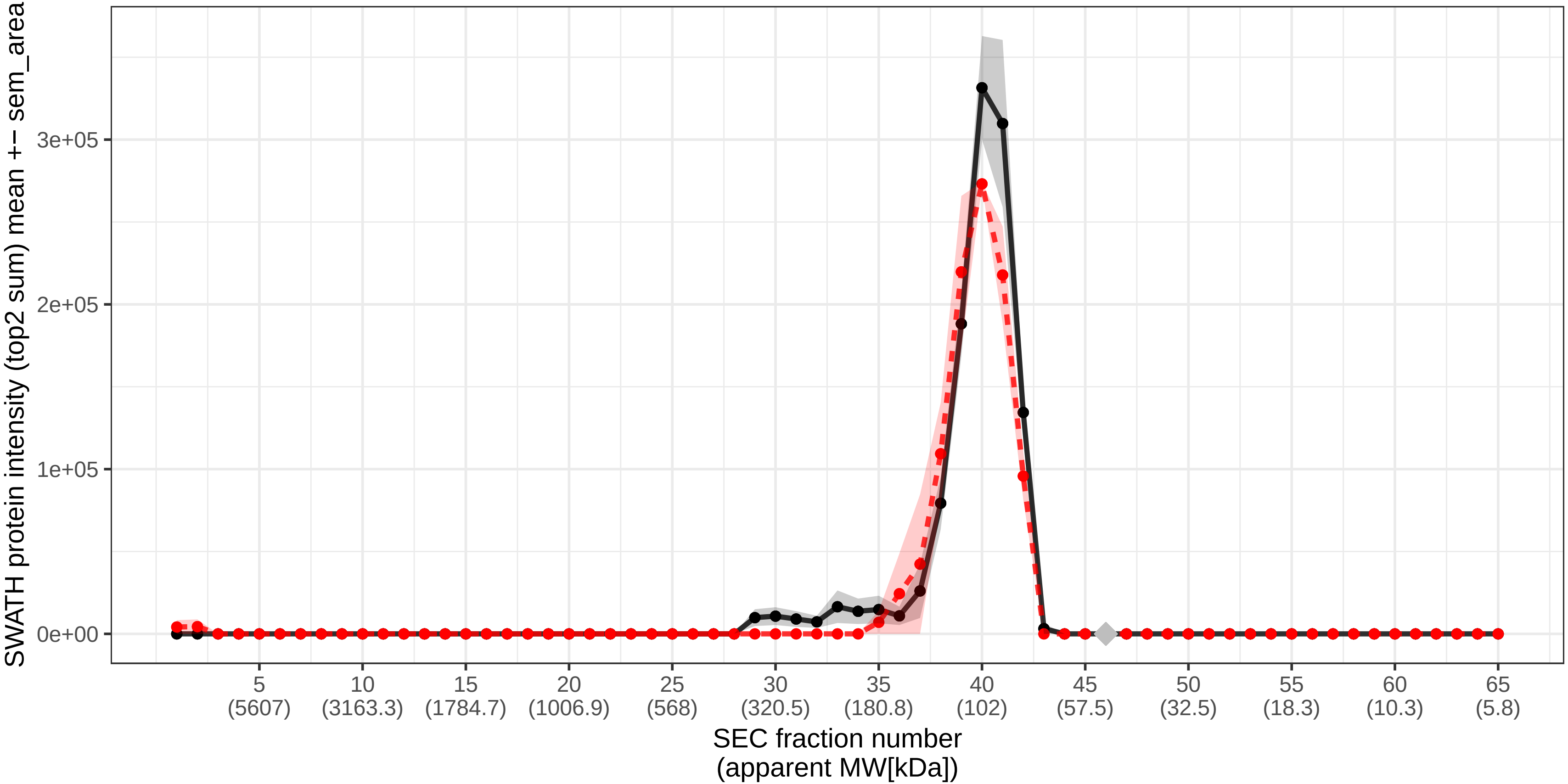

Supplement: Data S1. SEC-SWATH-MS Protein Chromatograms, Related to Figure 1 [file mmc6.zip › SECchrom_Q9BZG8_DPH1_HUMAN_DPH1_DPH2L_DPH2L1_OVCA1.pdf]
